# Stigmatising Attitudes Towards People With Depression, Bipolar Disorder, Borderline Personality, ADHD and Early and Long‐Term/Untreated Schizophrenia: Representative Survey of Australian Adults

**DOI:** 10.5694/mja2.70230

**Published:** 2026-06-23

**Authors:** Amy J. Morgan, Anna M. Ross, Gayle McNaught, Rachel Green, Nicola J. Reavley

**Affiliations:** ^1^ Melbourne School of Population and Global Health The University of Melbourne Carlton Victoria Australia; ^2^ SANE Carlton Victoria Australia

**Keywords:** attention deficit disorder with hyperactivity, bipolar and related disorders, depressive disorders, personality disorders, schizophrenia spectrum and other psychotic disorders

## Abstract

**Objectives:**

To examine the prevalence in Australia of stigmatising attitudes towards people with six different mental health conditions: depression, early and long‐term/untreated forms of schizophrenia, bipolar disorder, borderline personality disorder and attention‐deficit/hyperactivity disorder (ADHD).

**Design:**

Cross‐sectional population‐based survey using the probability‐based online panel Life in Australia. Participants responded to one of six vignettes describing a person with a mental health condition.

**Setting:**

Australia, 11–25 November 2024.

**Participants:**

Representative sample of 6032 adult residents of Australia.

**Main Outcome Measures:**

Proportions of participants who agreed or strongly agreed with 13 stigmatising attitudes and proportions who were definitely or probably unwilling to interact in five different social situations with the person in the vignette.

**Results:**

Stigmatising attitudes were generally lowest for depression and highest for long‐term schizophrenia and borderline personality disorder. Beliefs about unpredictability had the highest endorsement: 61.9% (95% confidence interval [CI], 58.2%–65.5%) for long‐term schizophrenia; 56.3% (95% CI, 52.5%–59.9%) for borderline personality disorder; 52.8% (95% CI, 49.0%–56.6%) for early schizophrenia; 50.7% (95% CI, 47.0%–54.4%) for bipolar disorder; 29.2% (95% CI, 25.9%–32.7%) for ADHD and 23.3% (95% CI, 20.2%–26.7%) for depression. Forcing treatment was endorsed by 25.9% (95% CI, 22.6%–29.5%) for early schizophrenia and 24.1% (95% CI, 21.0%–27.6%) for long‐term schizophrenia. For all conditions, at least 20% of participants did not agree that the person in the vignette was a person of worth, with agreement ranging from 78.5% (95% CI, 75.1%–81.6%) for early schizophrenia to 67.4% (95% CI, 63.8%–70.9%) for long‐term schizophrenia. There were high levels of unwillingness for the person in the vignette to marry into the family: ranging from 29.7% (95% CI, 26.4%–33.2%) for ADHD to 64.4% (95% CI, 60.7%–67.9%) for long‐term schizophrenia.

**Conclusions:**

Stigma related to mental health conditions remains prevalent in Australia and contributes to social and economic exclusion among those affected. Sustained action is needed across multiple sectors to address stigma, particularly towards conditions such as schizophrenia and borderline personality disorder, which are poorly understood within the community.

## Plain Language Summary

**The Known:** Stigma related to mental health can have profound negative impacts, leading to reluctance to seek help or disclose mental health problems, discrimination in personal relationships, healthcare and the workplace and poorer recovery outcomes.

**The New:** Stigmatising attitudes in the Australian population are prevalent, particularly towards people with schizophrenia and borderline personality disorder. Depression and attention‐deficit/hyperactivity disorder are relatively less stigmatised, with bipolar disorder associated with moderate levels of stigma.

**The Implications:** There is a need for continued action to reduce stigma within the Australian population so that people with mental health conditions can live full and meaningful lives.

## 1 Introduction

Mental health conditions are prevalent in the Australian population and are increasing in young people [1]. The burden of managing a mental health condition is compounded by stigma—the devaluation and discrediting of affected individuals. Public stigma refers to stereotypes, prejudice and discrimination held about a person with a mental health condition. Common stereotypes include beliefs that people with a mental health condition are dangerous, unpredictable and incompetent [2]. These have harmful impacts as they can lead to discrimination and social and economic exclusion in many areas of life, including making friends, receiving quality healthcare and finding and keeping employment. Reducing stigma is a priority in Australian mental health policy to improve well‐being in people with mental health conditions.

Research consistently shows that stigmatising attitudes vary by mental health condition. For example, in 2011, a representative sample of 6019 Australians aged 15 years or older completed questionnaires on stigmatising attitudes towards people with depression, schizophrenia, social anxiety disorder and posttraumatic stress disorder (PTSD) [3]. The proportion of respondents who endorsed dangerousness ranged from 15.5% for social anxiety disorder to 36.8% for chronic schizophrenia, and endorsement of unpredictability varied between 41.7% for social anxiety disorder and 76.2% for chronic schizophrenia. However, about 20% thought a person with social anxiety disorder, PTSD or depression could ‘snap out of it’, whereas only 11.6% believed this for a person with chronic schizophrenia. Willingness to interact also varied; nearly half were unwilling to have a person with chronic schizophrenia marry into the family versus only 17.2% for a person with PTSD. Since 2011, there have been significant increases in use of mental health services and mental health literacy activities [4]. The recent Australian Government investment into a national stigma and discrimination strategy reinforces the importance of assessing current attitudes to people with mental health conditions to inform delivery of anti‐stigma efforts [5].

There is a lack of good quality data on stigmatising attitudes of the Australian general public towards people with other mental health conditions, such as bipolar disorder, borderline personality disorder and attention‐deficit/hyperactivity disorder (ADHD). Data that exist are based on convenience samples, which may not accurately reflect the Australian population, or are from particular subpopulations, such as health professionals or university students [6, 7]. For bipolar disorder, most research worldwide has compared stigma to people with schizophrenia [8]. Studies find a lower proportion endorse dangerousness (about 30%) and a greater willingness to interact with people with bipolar disorder compared with schizophrenia [9, 10].

Research on stigma towards people with borderline personality disorder has focussed on the attitudes of mental health professionals, who endorse higher levels of stigma relative to other mental health conditions [11], including beliefs that these clients are manipulative, responsible for their behaviour and difficult to treat [12]. There is less research with the general public, who show much lower recognition of the disorder than other mental health conditions [13]. Existing research is based on convenience samples, which indicate greater stigma towards people with borderline personality disorder than towards those with common mental health problems or bipolar disorder, and similar stigma levels to those with schizophrenia [14, 15, 16].

Prescriptions for ADHD medication have grown considerably within Australia in recent years and may have been fuelled by ADHD content on social media platforms such as TikTok and greater awareness of the condition [17, 18]. While there is Australian research on the acceptability of a diagnosis of ADHD and treatment with medications [19], we lack data on whether Australians hold stigmatising views towards people with ADHD. A representative survey of German adults found relatively low levels of stigma compared with other mental health conditions [20]. Only 6% of the sample reported fearful reactions to an unlabelled vignette of an adult with ADHD, although 24% reported being annoyed by them and about 20% would not rent a room to them or introduce them to a friend. It is not known whether these findings would apply to the Australian public given the different socio‐cultural context.

This study aimed to examine the prevalence of stigmatising attitudes towards people with six different mental health conditions using a representative sample of Australian adults. We included depression and early and long‐term/untreated forms of schizophrenia for comparability with previous surveys and added three new conditions that lack rigorous Australian population‐level data on stigma: bipolar disorder, borderline personality disorder and ADHD.

## 2 Methods

### 2.1 Participants

Cross‐sectional survey data were collected as part of the 2024–2025 National Survey of Stigma and Discrimination [21]. Participants were recruited by the Social Research Centre through its probability‐based Life in Australia online panel [22]. Panel members are enrolled via random digit dialling or address‐based sampling, ensuring a known probability of selection and yielding more reliable and accurate estimates than non‐probability or opt‐in online panels. A stratified random sample was drawn from panellists on strata defined by age, gender, highest level of education and speaking a language other than English at home. Panel members were invited to participate via email and SMS (where available), with up to five reminders. Data were collected by the Social Research Centre during the period 11–25 November 2024 and provided in anonymised form. There were 9472 adults invited and online surveys were completed by 6032 Australian adults (response rate, 63.7%). A sample size of 6000 gives excellent precision of population proportions (margin of error ±1.3 percentage points overall and ±3.1 percentage points per vignette).

### 2.2 Measures

#### 2.2.1 Vignettes

Respondents were randomly assigned to read one of six vignettes, with the gender of the person in the vignette matched to respondent gender (Table 1). The vignettes described a person named Sam with the following conditions: (i) depression (*n* = 1008); (ii) early schizophrenia (*n* = 976); (iii) bipolar disorder (*n* = 1019); (iv) borderline personality disorder (*n* = 1011); (v) long‐term/untreated schizophrenia (*n* = 1005); (vi) ADHD (*n* = 1013). Vignettes were presented without diagnostic labels but satisfied the diagnostic criteria according to DSM‐IV and ICD‐10, and described a 24‐year‐old early in the course of illness before treatment had been sought (except for the vignette long‐term/untreated schizophrenia). The vignettes for depression, early schizophrenia and long‐term/untreated schizophrenia were previously used in the 2011 National Survey of Mental Health Literacy and Stigma [3]. The bipolar disorder and borderline personality disorder vignettes were taken from the National Survey of Mental Health‐Related Stigma and Discrimination [23] and the ADHD vignette was adapted from a research article by Speerforck et al. [24]. Where necessary, vignettes were adapted for length and consistency with the other vignettes (e.g., to include the name and age of the person).

**TABLE 1:** Vignettes.^a^

Mental health condition	Sam's situation
Depression	Sam is 24 years old. He has been feeling unusually sad and miserable for the last few weeks. Even though he is tired all the time, he has trouble sleeping nearly every night. Sam does not feel like eating and has lost weight. He cannot keep his mind on his work and puts off making decisions. Even day‐to‐day tasks seem too much for him. This has come to the attention of his boss, who is concerned about Sam's lowered productivity.
Early schizophrenia	Sam is 24 and lives at home with his parents. He has had a few temporary jobs since finishing school but is now unemployed. Over the last 6 months, he has stopped seeing his friends and has begun locking himself in his bedroom and refusing to eat with the family or to have a bath. His parents also hear him walking about his bedroom at night while they are in bed. Even though they know Sam is alone, they have heard him shouting and arguing as if someone else is there. When they try to encourage Sam to do more things, he whispers that he would not leave home because he is being spied upon by the neighbour. His parents realise he is not taking drugs because he never sees anyone or goes anywhere.
Bipolar disorder	Sam is 24 years old. In the past, there were times when he felt very sad and low without there being a specific reason for it. In contrast to this and to their usual behaviour, he is currently in an exceptionally good mood without any specific reason. He acts very impulsively and erratically. He speaks rapidly and tells others that he is having lots of new ideas and thoughts. He will often wake up earlier than usual but still feel bursting with energy. He sometimes manages without any sleep and still does not feel tired. Unusually for him, he has recently been spending more money than he can really afford.
Borderline personality disorder	Sam is 24 years old. His mood is unstable at times, and he regularly feels like nobody understands them. He sometimes has angry outbursts at his workplace and recently lost his job because of these problems. He enters new short‐term relationships quickly. He is terrified each new partner will leave them, which makes him lash out at his partner when he feels unloved. His relationships often seem to end with a fight or with arguments. He tends to find people either exceptionally admirable or terrible. He often feels like he is empty inside.
Long‐term/untreated schizophrenia	Sam is 38 years old. He is always on their own and is often seen sitting in the park talking to themselves. At times, he stands and moves his hands as if to communicate to someone in nearby trees. He rarely drinks alcohol. He speaks using uncommon and sometimes made‐up words. At times, he accuses shop owners of giving information about him to other people. His landlord complains that Sam will not let them clean the room, which is increasingly filled with glass objects. Sam says he is using these ‘to receive messages from space’. He has not worked a paid job for years.
Attention‐deficit/hyperactivity disorder	Sam is 24 years old. He often makes careless mistakes at work, often has difficulty remaining focussed during meetings, does not follow through on instructions and often fails to finish his work. At work and at home, he often does not seem to listen when spoken to directly and is easily distracted. He often loses things like his keys or mobile phone and is forgetful in daily activities like doing chores or running errands. He often fidgets or taps their hands or feet. He often talks excessively and blurts out an answer before a question has been completed. Several of these behaviours were already present when Sam was a child. These behaviours interfere with Sam's functioning at work and negatively affect his relationships with colleagues and friends.

^a^ Male version shown. Vignette gender (pronouns) was matched to participant gender.

#### 2.2.2 Personal Stigmatising Attitudes

After reading the vignette, respondents were presented with 13 randomly ordered questions on personally stigmatising attitudes. Nine of these were from the Personal Stigma Scale [25], an assessment of stigma with evidence for its validity [26]: (i) People with a problem like Sam's could snap out of it if they wanted; (ii) A problem like Sam's is a sign of personal weakness; (iii) Sam's problem is not a real medical illness; (iv) People with a problem like Sam's are dangerous; (v) It is best to avoid people with a problem like Sam's so that you don't develop this problem; (vi) People with a problem like Sam's are unpredictable; (vii) If I had a problem like Sam's I would not tell anyone; (viii) I would not employ someone if I knew they had a problem like Sam's; (ix) I would not vote for a politician if I knew they had suffered a problem like Sam's. An additional item was adapted from the National Survey of Mental Health‐Related Stigma and Discrimination [23]: (x) People with a problem like Sam's should be forced into treatment even if they don't want to. Finally, three items assessing attitudes about the social worth of people with mental health conditions were adapted from the validated Empowerment Scale [27]: (xi) I feel people with a problem like Sam's are persons of worth, at least on an equal basis with others; (xii) I see people with a problem like Sam's as capable people; (xiii) People with a problem like Sam's are able to do things as well as most other people. Each of the 13 items was rated on a 5‐point agreement scale. Responses were dichotomised into agree or strongly agree (4 or 5) versus strongly disagree, disagree, or neither agree nor disagree (1–3). To enable examination of participant characteristics associated with stigma without relying on numerous item‐level tests, an exploratory factor analysis of a polychoric correlation matrix (principal factor extraction, oblimin rotation) was conducted to identify underlying dimensions (see Figures S1 and S2, Table S3). This supported a two‐factor solution based on parallel analysis, eigenvalues and scree plot; polychoric correlations were estimated using all available data (pairwise present). Mean scores were calculated on two dimensions of personal stigma: representing beliefs that Sam is dangerous, unpredictable and incapable (items iv, vi, viii, ix, x, xii, xiii) and representing beliefs that Sam is weak rather than sick (items i, ii, iii and v). Internal consistency, as measured with McDonald's *ω*, was good for both dimensions (0.83 for dangerous, unpredictable and incapable and 0.85 for weak not sick).

#### 2.2.3 Desire for Social Distance

Willingness to interact with the person in the vignette was measured with the Social Distance Scale [28], a validated measure of stigma [26, 29]. This includes five items assessing willingness to (i) move next door to Sam; (ii) spend an evening socialising with Sam; (iii) make friends with Sam; (iv) work closely with Sam on a job; (v) have Sam marry into their family. Each item was rated on a 4‐point scale: 1, definitely willing; 2, probably willing; 3, probably unwilling; 4, definitely unwilling. Responses were dichotomised into probably or definitely unwilling (3 or 4) versus probably or definitely willing (1 or 2). A mean score was also calculated, ranging from 1 to 4, with higher scores indicating greater stigma (*ω* = 0.94).

#### 2.2.4 Demographics

The following sociodemographic information was collected: state or territory; capital city or rest of state or territory; age; gender (man or male, woman or female, non‐binary, or a different term); citizenship status; Aboriginal and Torres Strait Islander status; country of birth; language spoken at home; highest level of primary/secondary school completed; level of the highest educational qualification; employment status; marital status; and family composition.

### 2.3 Statistical Analysis

Data were weighted to account for the probability of selection into the panel and the survey study itself with adjustment for response or non‐response. This weight was then adjusted to satisfy population benchmarks for age, gender, language other than English spoken at home, highest level of education, state or territory, geographic location (all based on the 2021 Census), supplemented by the latest demographic statistics [30] and the number of adults in the household (based on the 2020–2021 National Health Survey [31]). The weighted data were analysed using percentage frequencies and 95% confidence intervals. Independent estimates with non‐overlapping confidence intervals provide strong evidence that they are significantly different at *p* < 0.05. There were very small rates of missing responses (0.2%–0.4% for questions on personal stigmatising attitudes and 0.5%–1.4% for questions on desire for social distance), which were ignored per analysis. Multiple linear regression models were conducted on mean stigma scores to evaluate significant predictors of stigma. All analyses were conducted using StataSE 18.

### 2.4 Ethics Statement

The study received ethics approval from the University of Melbourne Human Research Ethics Committee (ID30886).

## 3 Results

**FIGURE 1:**
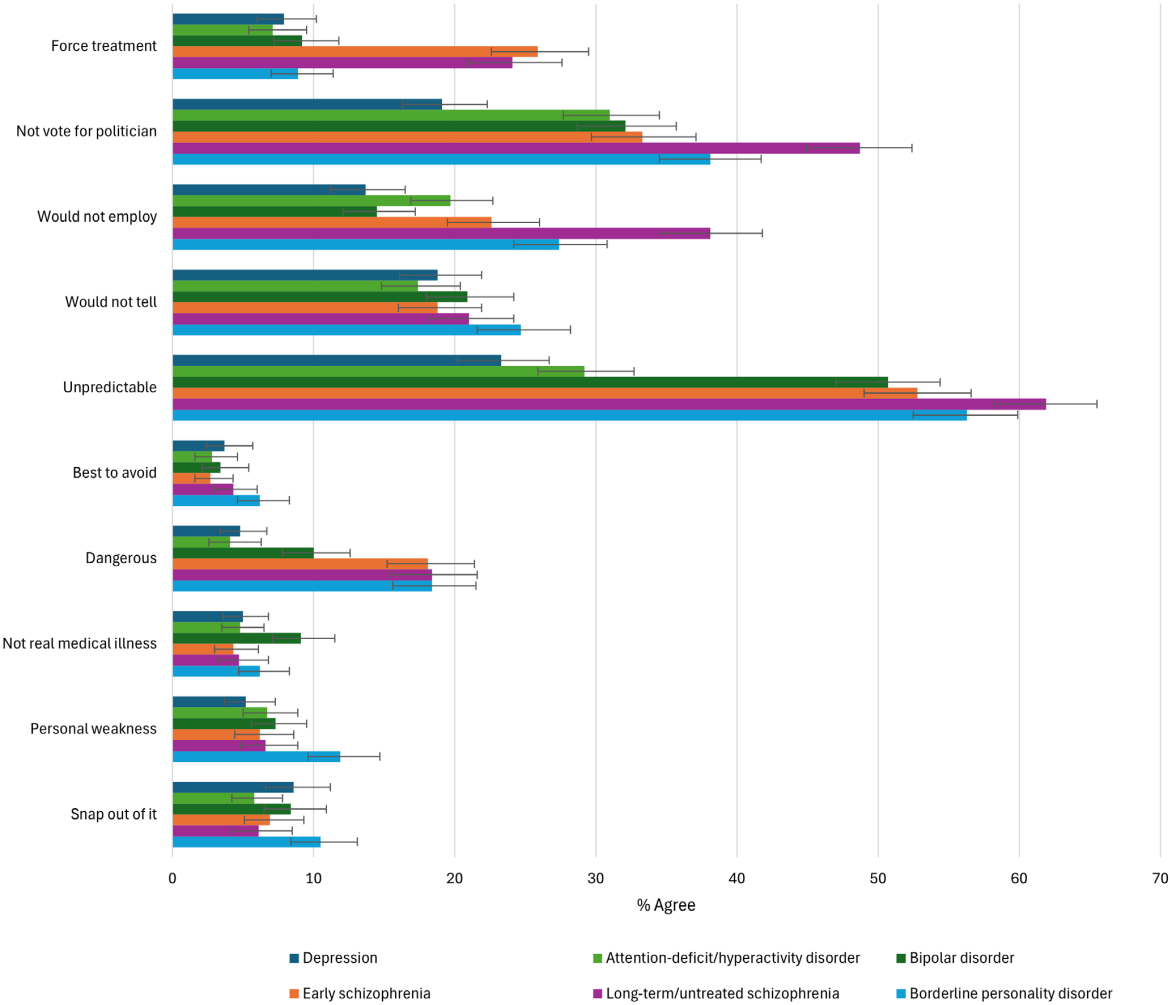
Percentage of participants who agreed or strongly agreed with each stigmatising attitude per mental health condition.

Participant characteristics are shown in Table 2. Figure 1 shows the percentages of participants who agreed or strongly agreed with each stigmatising attitude per mental health condition. Full details of each response category are provided in Table S1. As can be seen in Figure 1, level of agreement varied by attitude and mental health condition. Stigmatising attitudes were generally highest for long‐term schizophrenia and borderline personality disorder. Several beliefs were endorsed by only a minority of participants for all conditions, including the beliefs that Sam could snap out of the problem, the problem is a sign of personal weakness and the problem is not a real medical illness. In contrast, there was much greater agreement regarding unpredictability, with more than 50% agreeing that people with long‐term/untreated schizophrenia, borderline personality disorder and early schizophrenia were unpredictable. More than a quarter agreed that people with early or long‐term schizophrenia should be forced into treatment even if they don't want to, whereas this was rarely endorsed for the other conditions.

**TABLE 2:** Participant characteristics (*N* = 6032).

Characteristic	*n*	Weighted percentage
Age group		
18–34 years	1186	30.1
35–64 years	3449	48.2
65+ years	1394	21.8
Gender		
Female/woman	3580	48.3
Male/man	2391	50.3
Other (non‐binary or use a different term)	58	1.3
Highest level of education		
Below bachelor level	2910	66.3
Bachelor degree or above	3068	33.7
Employment status		
In paid employment	3776	63.6
Not in paid employment	2246	36.4
Marital status		
Married or in de facto relationship	3803	64.7
Not married	2202	35.3
Family composition		
Couple family with no children	1879	33.4
Couple family with no children under 15 years	493	9.0
Couple family with children under 15 years	1104	19.7
One parent family with no children under 15 years	239	4.4
One parent family with children under 15 years	301	5.2
Other family	352	10.1
Not applicable	1515	18.2
State or territory		
New South Wales	1869	31.8
Victoria	1557	25.7
Queensland	1172	20.1
South Australia	519	7.1
Western Australia	578	10.3
Tasmania	165	2.2
Northern Territory	33	0.9
Australian Capital Territory	139	1.8
Region		
Capital city	4152	67.0
Rest of the state or territory	1861	33.0
Country of birth		
Australia or English‐speaking country	5040	80.7
Non‐English‐speaking country	985	19.3
Language spoken at home		
English	5048	75.8
Other	979	24.2
Aboriginal or Torres Strait Islander status		
Yes	128	2.5
No	5887	97.5

*Note:* Responses may not sum to 6032 due to missing data on some participant characteristics.

**FIGURE 2:**
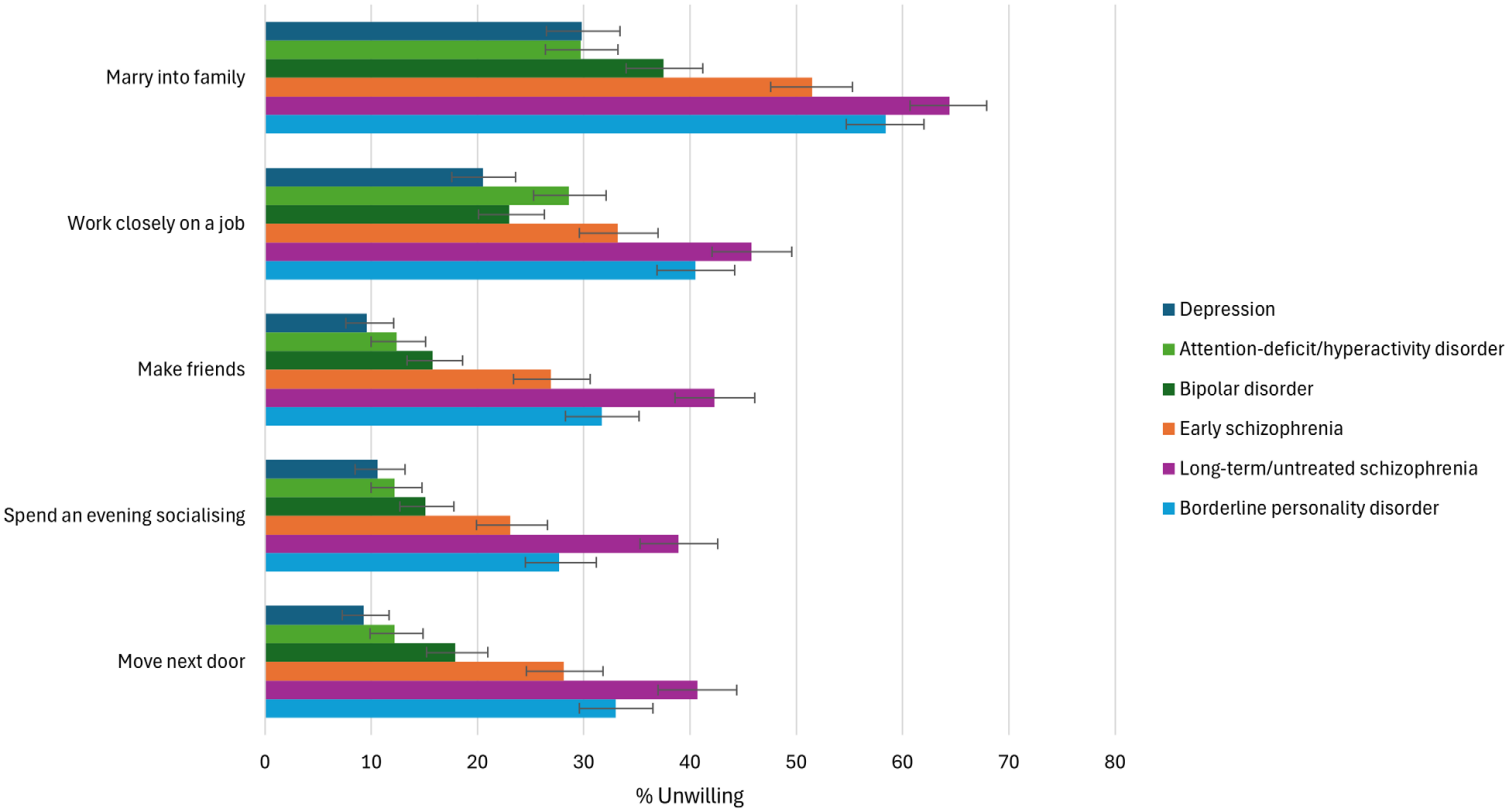
Data from the Social Distance Scale on unwillingness to interact with the person in the vignette per mental health condition.

Figure 2 presents the data from the Social Distance Scale on unwillingness to interact with the person in the vignette per mental health condition. Full details of each response category are provided in Table S2. Unwillingness varied per condition and was highest for long‐term/untreated schizophrenia and borderline personality disorder. At least 30% of respondents reported being unwilling for Sam to marry into their family, rising to 64% for the long‐term/untreated schizophrenia vignette. Mean scores were calculated per vignette and from lowest to highest were: depression *M* = 1.88 (95% CI, 1.84–1.93), ADHD *M* = 1.90 (95% CI, 1.86–1.95), bipolar disorder *M* = 2.02 (95% CI, 1.98–2.07), early schizophrenia *M* = 2.21 (95% CI, 2.17–2.26), borderline personality disorder *M* = 2.33 (95% CI, 2.28–2.37) and long‐term/untreated schizophrenia *M* = 2.47 (95% CI, 2.42–2.52).

Data on the three items assessing social worth are presented in Table 3. For all conditions, the majority of respondents agreed that people with a problem like Sam's are persons of worth, although there were at least 20% who did not agree. There were lower levels of agreement that they were able to do things as well as most other people, particularly for the schizophrenia vignettes.

**TABLE 3:** Agreement with attitudes about the social worth of people with mental health conditions.

	Percentage of participants who agree (95% CI)		
	Do things as well	Capable people	Persons of worth
Depression	47.6 (43.9–51.3)	63.9 (60.2–67.4)	78.1 (74.8–81.1)
Early schizophrenia	39.7 (36.0–43.4)	49.4 (45.6–53.2)	78.5 (75.1–81.6)
Bipolar disorder	59.0 (55.3–62.6)	67.7 (64.2–71.0)	78.1 (74.9–81.1)
Borderline personality disorder	54.6 (50.9–58.2)	57.5 (53.8–61.0)	73.5 (70.1–76.7)
Long‐term schizophrenia	28.2 (24.9–31.7)	32.1 (28.7–35.7)	67.4 (63.8–70.9)
Attention‐deficit/hyperactivity disorder	54.0 (50.3–57.7)	62.4 (58.8–66.0)	75.3 (71.9–78.5)

Table 4 presents the findings from multiple linear regression models on characteristics associated with personal stigma and desire for social distance. Controlling for vignette, higher levels of stigma were consistently associated with male gender, older age, speaking a language other than English at home, no mental health problems in the past year and not knowing someone with a mental health problem in the past year.

**TABLE 4:** Sociodemographic characteristics associated with personal stigmatising attitudes and desire for social distance based on multiple regression models.

Predictor	Personal stigma—weak not sick (*n* = 5897)		Personal stigma—dangerous, unpredictable and incapable (*n* = 5899)		Social distance (*n* = 5862)	
	*b* (95% CI)	*p*	*b* (95% CI)	*p*	*b* (95% CI)	*p*
Vignette						
Depression (reference category)	—		—		—	
Early schizophrenia	−0.09 (−0.16 to −0.02)	0.009	0.51 (0.45 to 0.58)	< 0.001	0.32 (0.26, 0.38)	< 0.001
Bipolar disorder	0.12 (0.05 to 0.19)	0.001	0.19 (0.13 to 0.26)	< 0.001	0.13 (0.07, 0.19)	< 0.001
Borderline personality disorder	0.25 (0.18 to 0.32)	< 0.001	0.38 (0.32 to 0.44)	< 0.001	0.43 (0.37, 0.50)	< 0.001
Long‐term schizophrenia	−0.05 (−0.12 to 0.02)	0.168	0.71 (0.64 to 0.77)	< 0.001	0.56 (0.50, 0.63)	< 0.001
Attention‐deficit/hyperactivity disorder	0.02 (−0.04 to 0.09)	0.497	0.09 (0.02 to 0.15)	0.012	0.01 (−0.05, 0.07)	0.703
Gender						
Male/man (reference category)	—		—		—	
Female/woman	−0.18 (−0.22 to −0.13)	< 0.001	−0.20 (−0.24 to −0.17)	< 0.001	−0.21 (−0.25, −0.17)	< 0.001
Other (non‐binary or a different term)	−0.38 (−0.54 to −0.22)	< 0.001	−0.38 (−0.57 to −0.19)	< 0.001	−0.29 (−0.47, −0.12)	0.001
Age group						
18–34 years (reference category)	—		—		—	
35–64 years	0.09 (0.04 to 0.14)	0.001	0.09 (0.05 to 0.14)	< 0.001	0.04 (−0.01, 0.09)	0.106
65+ years	0.11 (0.04 to 0.17)	0.001	0.15 (0.10 to 0.21)	< 0.001	0.09 (0.03, 0.15)	0.004
Region						
Capital city (reference category)	—		—		—	
Rest of state or territory	−0.03 (−0.07 to 0.01)	0.193	−0.00 (−0.04 to 0.04)	0.987	−0.03 (−0.08, 0.02)	0.125
Highest education						
Below bachelor level (reference category)	—		—		—	
Bachelor degree or above	−0.06 (−0.10 to −0.01)	0.009	0.01 (−0.03 to 0.04)	0.777	0.07 (0.03, 0.11)	< 0.001
Language spoken at home						
Language other than English (reference category)	—		—		—	
English only	−0.40 (−0.47 to −0.34)	< 0.001	−0.15 (−0.21 to −0.10)	< 0.001	−0.14 (−0.19, −0.08)	< 0.001
Mental health problem in past 12 months						
No (reference category)	—		—		—	
Yes	−0.16 (−0.21 to −0.12)	< 0.001	−0.15 (−0.19 to −0.11)	< 0.001	−0.15 (−0.19, −0.11)	< 0.001
Knew someone with mental health problem in past 12 months						
No (reference category)	—		—		—	
Yes	−0.28 (−0.33 to −0.23)	< 0.001	−0.13 (−0.17 to −0.09)	< 0.001	−0.16 (−0.20, −0.12)	< 0.001
*R* ^2^	0.20		0.23		0.20	

## 4 Discussion

This study aimed to assess the level of stigma within the Australian population towards individuals with mental health conditions. Our large representative sample confirms that stigma is still prevalent but levels vary by mental health condition. Of the six conditions examined, depression was the least stigmatised and schizophrenia and borderline personality disorder were the most severely stigmatised. Overall, our findings present a mixed picture of stigma towards people with mental health conditions. Thirty years ago, many Australians believed that mental health conditions were caused by a weakness of character and were not genuine medical illnesses [32], but these beliefs were only endorsed by a small minority in 2024. Beliefs about dangerousness are also lower than in the 2011 survey [3], although a substantial proportion of participants endorsed neutral responses. This may reflect increasingly nuanced attitudes towards people with mental health conditions, where judgements are based on the individual and their circumstances rather than applied as blanket stereotypes. Of concern are the high endorsements of unpredictability (even for less stigmatised conditions like depression), the significant proportions who wanted to avoid having contact with the people described in the vignettes, the significant proportions who did not agree that they were persons of worth, and the 20%–25% of respondents who would not tell anyone if they had the same condition. These beliefs indicate problematic levels of stigma remain in the community, negatively impacting a range of life domains and presenting a barrier to seeking support from health professionals and social networks to aid in recovery.

This study provides novel insights on stigma towards mental health conditions that lack robust population data. To our knowledge, this is the first nationally representative study to compare stigma towards people with borderline personality disorder, bipolar disorder and ADHD that has used probability‐based sampling. There were high levels of stigma towards people with borderline personality disorder, consistent with previous research with members of the public and health professionals [11, 15]. As this condition is less well known than others, participants may have been responding to the description of symptoms, which focused on interpersonal difficulties, rather than relying on stereotypes associated with a diagnostic label. Indeed, some evidence suggests that stigmatising responses such as blame and anger are lower when borderline personality disorder symptoms are accompanied by a diagnostic label, as the label can provide an explanation for behaviour that might otherwise be seen as personally controllable [16].

Stigma towards the ADHD vignette was similar to that of the depression vignette, suggesting Australians tend to hold relatively non‐stigmatising views on ADHD, consistent with German data [20]. The exception was a small increase in stigma relative to depression on willingness to work closely with a person with ADHD or vote for a politician with ADHD, which may reflect the vignette's focus on work impairments. Stigma towards people with bipolar disorder was similar to that for those with depression, except for higher levels of unpredictability and not voting for a politician with the condition. Of note, the bipolar disorder vignette had the highest endorsement of beliefs about capability and doing things as well as other people, which may reflect a perceived link to creative genius [33].

These findings reinforce the need for action outlined in the national strategy to reduce mental health‐related stigma and discrimination [5]. Given the complexity of the issue, the strategy details a comprehensive approach to reducing stigma across different levels and domains. These include legislative and health system reforms, improving support for the health and mental health workforces, expanding the lived experience peer workforce, and education and training for key cohorts of people who have frequent contact with or significant impact on the lives of people with mental health conditions. For health professionals, the strategy recommends strengthening pre‐service training and ongoing professional development that includes a human rights framing, and activities that include contact with people with mental health conditions who are in recovery, which counter‐balance perceptions of chronic impairment or incapacity.

### 4.1 Limitations

This study has several limitations that should be acknowledged. Vignettes are the standard approach to measuring stigmatising attitudes but may not accurately reflect attitudes to real‐life situations or individuals with different symptom presentations or impairments. Furthermore, attitudes may be different towards vignettes that describe a person living well in the community after undergoing treatment. While our data were weighted to match population benchmarks, the 63.7% response rate raises the possibility of non‐response bias. Although knowing someone with a mental health condition was measured, population benchmarks for this characteristic are unavailable, so we cannot assess whether our sample is representative in this respect and some bias may remain. Finally, responses to the schizophrenia vignettes may have been influenced by extensive media coverage at the time of the coronial inquest into the Bondi Junction mass stabbing, which often focussed on the perpetrator's untreated schizophrenia [34].

## 5 Conclusion

In conclusion, stigma related to mental health remains prevalent in Australia and may contribute to social and economic exclusion among those affected. There is a need for sustained action across multiple sectors to address stigma, particularly towards conditions such as schizophrenia and borderline personality disorder, which are poorly understood within the community.

## Author Contributions

**Amy J. Morgan:** conceptualisation, data curation, formal analysis, methodology, writing – original draft. **Nicola J. Reavley:** conceptualisation, data curation, formal analysis, methodology, project administration, supervision, writing – review and editing. **Anna M. Ross:** conceptualisation, writing – review and editing. **Rachel Green:** funding acquisition, project administration, conceptualisation, writing – review and editing. **Gayle McNaught:** conceptualisation, project administration, writing – review and editing. Amy Morgan and Nicola Reavley had full access to all the data in the study and had final responsibility to submit for publication.

## Funding

The 2024–2025 National Survey of Stigma and Discrimination was funded by SANE. Anna Ross receives fellowship funding from the National Health and Medical Research Council (Investigator Grant No. 2025181), Amy Morgan receives funding from Mental Health First Aid International and Veski, and NR receives funding from SANE. Nicola Reavley, Anna Ross, Gayle McNaught and Rachel Green receive funding or salaries from SANE. The funding sources played no role in study design, data collection, analysis or interpretation, reporting or publication, except for the scholarly collaborations of Gayle McNaught and Rachel Green.

## Disclosure

Not commissioned; externally peer reviewed.

## Conflicts of Interest

Amy Morgan is co‐founder of Mental Ill‐health Stigma Researchers Network Australia and Anna Ross is on the organising committee of this network.

## Supporting information


**Data S1:** mja270230‐sup‐0001‐supinfo.docx.

## Data Availability

Data are available upon reasonable request.
