# Supplementary material for: Stigmatising Attitudes Towards People With Depression, Bipolar Disorder, Borderline Personality, ADHD and Early and Long‐Term/Untreated Schizophrenia: Representative Survey of Australian Adults
Source: Med J Aust. 2026 Jun 23;224(6):e70230. doi: 10.5694/mja2.70230 (PMC13288448; doi:10.5694/mja2.70230)
Supplement: Supplementary file 1 — Data S1: mja270230‐sup‐0001‐supinfo.docx. [file MJA2-224-0-s001.docx]

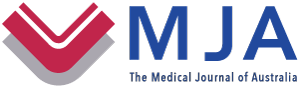


Supporting Information

Supplementary material

**This appendix was part of the submitted manuscript and has been peer reviewed.
It is posted as supplied by the authors.**

Appendix to: Morgan A, Ross A, McNaught G, et al. Stigmatising Attitudes Towards People With Depression, Bipolar Disorder, Borderline Personality, ADHD and Early and Long-Term/Untreated Schizophrenia: Representative Survey of Australian Adults. *Med J Aust* 2026; doi: 10.5694/mja2.00000.

Table S1. Personal stigma item responses per vignette

|  | Strongly disagree  % [95% CI] | Disagree  % [95% CI] | Neither  % [95% CI] | Agree  % [95% CI] | Strongly agree  % [95% CI] |
| --- | --- | --- | --- | --- | --- |
| Depression |  |  |  |  |  |
| snap out of it | 33.7 [30.3,37.2] | 41.2 [37.6,44.9] | 16.5 [13.9,19.5] | 7.4 [5.5,9.9] | 1.2 [0.6,2.6] |
| personal weakness | 44.0 [40.4,47.7] | 40.8 [37.3,44.5] | 10.0 [7.9,12.5] | 4.2 [2.8,6.1] | 1.0 [0.5,2.2] |
| not real medical illness | 42.3 [38.7,46] | 39.3 [35.7,42.9] | 13.5 [11.0,16.4] | 3.9 [2.8,5.5] | 1.0 [0.5,2.2] |
| dangerous | 36.0 [32.5,39.6] | 38.4 [34.9,42] | 20.9 [17.9,24.2] | 3.7 [2.5,5.4] | 1.1 [0.5,2.4] |
| best to avoid | 47.0 [43.3,50.7] | 39 [35.5,42.7] | 10.2 [8.2,12.8] | 2.1 [1.2,3.6] | 1.7 [0.9,3.2] |
| unpredictable | 12.8 [10.5,15.4] | 27.3 [24.3,30.6] | 36.6 [33.1,40.3] | 22.1 [19.1,25.4] | 1.2 [0.6,2.5] |
| would not tell | 13.6 [11.3,16.4] | 42.6 [39,46.3] | 25.0 [21.9,28.3] | 15.6 [13.1,18.5] | 3.2 [2.0,4.9] |
| would not employ | 21.7 [18.8,24.9] | 35.0 [31.6,38.6] | 29.6 [26.3,33.1] | 12 [9.7,14.7] | 1.7 [0.9,3.1] |
| would not vote for | 21.8 [18.8,25.1] | 33.4 [30.1,36.9] | 25.7 [22.6,29.0] | 16.1 [13.5,19.2] | 3.0 [2.0,4.5] |
| force into treatment | 26.3 [23.3,29.7] | 44.9 [41.2,48.6] | 20.9 [18.0,24.2] | 6.5 [4.9,8.7] | 1.3 [0.6,2.7] |
| persons of worth | 3.2 [2.1,5.0] | 4.2 [2.9,6.1] | 14.5 [12.0,17.3] | 46.9 [43.2,50.6] | 31.2 [27.9,34.6] |
| capable people | 1.1 [0.5,2.3] | 7.9 [6.0,10.2] | 27.2 [24.0,30.6] | 49.1 [45.4,52.8] | 14.7 [12.3,17.6] |
| able to do things | 4.5 [3.2,6.3] | 21.9 [19.0,25.1] | 26.0 [23.0,29.4] | 37.3 [33.8,40.9] | 10.3 [8.2,12.9] |
|  |  |  |  |  |  |
| Early schizophrenia |  |  |  |  |  |
| snap out of it | 38.5 [34.9,42.3] | 40.6 [36.9,44.4] | 13.9 [11.4,17] | 6.4 [4.7,8.7] | 0.5 [0.2,1.3] |
| personal weakness | 41.6 [37.9,45.4] | 40.9 [37.2,44.7] | 11.3 [9,14.0] | 5.2 [3.6,7.5] | 1.0 [0.5,2.2] |
| not real medical illness | 52 [48.2,55.8] | 35.8 [32.3,39.5] | 7.9 [5.9,10.5] | 3.4 [2.2,5.1] | 0.9 [0.4,1.9] |
| dangerous | 8.7 [6.8,11.1] | 27.3 [24.2,30.8] | 45.8 [42,49.6] | 16.5 [13.7,19.6] | 1.7 [0.8,3.3] |
| best to avoid | 48.0 [44.2,51.8] | 39.0 [35.4,42.8] | 10.3 [8.1,13] | 2.4 [1.4,4.0] | 0.3 [0.1,0.8] |
| unpredictable | 2.3 [1.3,4.0] | 11.5 [9.3,14.1] | 33.4 [29.9,37.1] | 47.7 [43.9,51.5] | 5.1 [3.7,7.0] |
| would not tell | 14.2 [11.7,17.2] | 40.7 [37,44.5] | 26.3 [23,29.8] | 17.1 [14.4,20.1] | 1.7 [1.0,2.9] |
| would not employ | 7.8 [6.0,10.0] | 28.9 [25.6,32.5] | 40.7 [37,44.5] | 21.4 [18.4,24.8] | 1.1 [0.6,2.1] |
| would not vote for | 8.5 [6.6,10.8] | 25.4 [22.2,28.8] | 32.9 [29.4,36.5] | 25.1 [21.9,28.7] | 8.2 [6.2,10.7] |
| force into treatment | 7.2 [5.5,9.4] | 30.6 [27.2,34.2] | 36.3 [32.7,40.1] | 23.3 [20.1,26.8] | 2.6 [1.5,4.4] |
| persons of worth | 1.3 [0.7,2.5] | 4.7 [3.3,6.7] | 15.4 [12.8,18.5] | 53.2 [49.4,57] | 25.3 [22.2,28.7] |
| capable people | 1.6 [0.9,3.0] | 12.7 [10.2,15.7] | 36.3 [32.7,40.1] | 43.0 [39.3,46.8] | 6.4 [4.9,8.4] |
| able to do things | 3.1 [2.0,4.8] | 24.9 [21.7,28.3] | 32.3 [28.8,36.1] | 35.0 [31.4,38.7] | 4.7 [3.3,6.5] |
|  |  |  |  |  |  |
| Bipolar disorder |  |  |  |  |  |
| snap out of it | 29.3 [26.1,32.8] | 41.5 [37.9,45.2] | 20.7 [17.8,24.0] | 7.7 [5.9,10.1] | 0.7 [0.3,1.8] |
| personal weakness | 37.9 [34.4,41.6] | 39.0 [35.4,42.6] | 15.8 [13.2,18.8] | 6.0 [4.5,8.1] | 1.3 [0.6,2.5] |
| not real medical illness | 34.0 [30.5,37.6] | 39.8 [36.2,43.5] | 17.2 [14.6,20.2] | 7.9 [6.1,10.3] | 1.1 [0.5,2.4] |
| dangerous | 18.8 [16,21.8] | 41.8 [38.2,45.6] | 29.5 [26.2,33] | 9.1 [7.1,11.6] | 0.9 [0.3,2.2] |
| best to avoid | 43.0 [39.4,46.7] | 39.7 [36.1,43.4] | 13.9 [11.5,16.8] | 2.6 [1.5,4.4] | 0.8 [0.3,2.1] |
| unpredictable | 3.0 [1.9,4.7] | 14.0 [11.6,16.8] | 32.3 [28.9,36.0] | 46.1 [42.4,49.8] | 4.6 [3.3,6.4] |
| would not tell | 10.7 [8.6,13.3] | 37.7 [34.2,41.3] | 30.7 [27.3,34.2] | 17.3 [14.7,20.3] | 3.6 [2.3,5.6] |
| would not employ | 15.6 [12.9,18.7] | 37.4 [33.8,41.0] | 32.6 [29.2,36.1] | 13.1 [10.8,15.8] | 1.4 [0.8,2.5] |
| would not vote for | 11.1 [8.9,13.7] | 26.9 [23.7,30.3] | 29.9 [26.6,33.4] | 23.1 [20.1,26.4] | 9.0 [7.0,11.5] |
| force into treatment | 23.9 [20.9,27.3] | 40.4 [36.8,44.1] | 26.4 [23.2,29.9] | 8.1 [6.2,10.5] | 1.1 [0.5,2.4] |
| persons of worth | 1.4 [0.6,2.8] | 4.1 [2.9,5.8] | 16.4 [13.8,19.4] | 47.6 [43.9,51.4] | 30.5 [27.2,34.0] |
| capable people | 2.0 [1.1,3.5] | 6.0 [4.5,8.0] | 24.3 [21.3,27.5] | 55.5 [51.7,59.1] | 12.2 [10.0,14.9] |
| able to do things | 2.4 [1.4,4.1] | 13.4 [11.1,16.1] | 25.2 [22.1,28.5] | 48.6 [44.9,52.3] | 10.5 [8.4,13.0] |
|  |  |  |  |  |  |
| Borderline Personality disorder |  |  |  |  |  |
| snap out of it | 26.9 [23.8,30.3] | 42.7 [39.1,46.3] | 19.9 [17,23.2] | 9.6 [7.5,12.2] | 0.9 [0.5,1.8] |
| personal weakness | 29.9 [26.7,33.4] | 39.8 [36.3,43.5] | 18.4 [15.6,21.5] | 10.2 [8.1,12.9] | 1.7 [0.9,2.9] |
| not real medical illness | 30.6 [27.3,34.1] | 42.7 [39.1,46.4] | 20.5 [17.6,23.7] | 5.7 [4.2,7.8] | 0.5 [0.2,1.2] |
| dangerous | 11.0 [8.9,13.5] | 31.2 [27.9,34.7] | 39.4 [35.8,43.1] | 17.4 [14.7,20.4] | 1.0 [0.5,2.2] |
| best to avoid | 28.2 [25.1,31.5] | 46.2 [42.5,49.9] | 19.4 [16.5,22.6] | 5.4 [4.0,7.4] | 0.8 [0.3,2.0] |
| unpredictable | 1.1 [0.6,2.3] | 11.6 [9.4,14.1] | 31.0 [27.7,34.6] | 50.7 [47,54.4] | 5.5 [4.0,7.5] |
| would not tell | 8.6 [6.8,10.8] | 41.8 [38.2,45.5] | 24.8 [21.8,28.2] | 20.1 [17.3,23.3] | 4.6 [3.2,6.7] |
| would not employ | 8.8 [6.9,11.2] | 27.4 [24.2,30.8] | 36.4 [32.9,40.0] | 23.8 [20.8,27.1] | 3.6 [2.4,5.4] |
| would not vote for | 7.8 [6.0,10.0] | 22.5 [19.6,25.8] | 31.6 [28.2,35.1] | 28.6 [25.4,32.1] | 9.4 [7.5,11.8] |
| force into treatment | 19.1 [16.4,22.2] | 44.2 [40.5,47.9] | 27.8 [24.5,31.3] | 7.5 [5.7,9.8] | 1.4 [0.8,2.7] |
| persons of worth | 0.7 [0.3,1.6] | 4.4 [3.1,6.4] | 21.4 [18.4,24.6] | 53.4 [49.7,57.1] | 20.1 [17.3,23.3] |
| capable people | 0.9 [0.4,1.8] | 8.4 [6.6,10.6] | 33.3 [30.0,36.9] | 49.8 [46.1,53.5] | 7.7 [5.9,9.9] |
| able to do things | 1.4 [0.8,2.6] | 16.0 [13.5,18.8] | 28.0 [24.8,31.4] | 44.2 [40.6,47.9] | 10.4 [8.2,13.1] |
|  |  |  |  |  |  |
| Long-term schizophrenia |  |  |  |  |  |
| snap out of it | 42.2 [38.5,45.9] | 36.8 [33.3,40.5] | 14.9 [12.5,17.8] | 4.8 [3.3,6.8] | 1.3 [0.5,3.2] |
| personal weakness | 40.6 [37.0,44.3] | 38.5 [34.9,42.2] | 14.3 [11.8,17.2] | 4.6 [3.2,6.6] | 2.0 [1.1,3.6] |
| not real medical illness | 50.8 [47.0,54.5] | 36.1 [32.5,39.8] | 8.4 [6.5,10.9] | 3.2 [2.1,5.0] | 1.5 [0.8,2.9] |
| dangerous | 8.0 [6.1,10.5] | 25.9 [22.8,29.2] | 47.7 [44.0,51.5] | 16.1 [13.3,19.2] | 2.3 [1.3,3.9] |
| best to avoid | 43.8 [40.2,47.6] | 38.5 [34.9,42.2] | 13.5 [11.1,16.3] | 2.9 [1.9,4.3] | 1.4 [0.7,2.7] |
| unpredictable | 1.7 [1.0,3.0] | 7.0 [5.3,9.3] | 29.3 [26,32.8] | 51.5 [47.8,55.3] | 10.4 [8.3,12.9] |
| would not tell | 12.1 [9.8,14.8] | 37 [33.5,40.8] | 29.9 [26.6,33.4] | 16.8 [14.2,19.8] | 4.2 [2.9,5.9] |
| would not employ | 4.6 [3.3,6.5] | 19.9 [17.1,23.1] | 37.3 [33.8,41.0] | 31.6 [28.3,35.2] | 6.4 [4.8,8.6] |
| would not vote for | 5.9 [4.3,8.0] | 18.6 [15.9,21.6] | 26.9 [23.7,30.3] | 32 [28.6,35.6] | 16.6 [13.9,19.8] |
| force into treatment | 10.7 [8.5,13.4] | 29.3 [26.1,32.7] | 35.9 [32.3,39.6] | 21 [18,24.2] | 3.2 [2.0,5.0] |
| persons of worth | 1.9 [1.2,3.1] | 7.0 [5.3,9.1] | 23.7 [20.5,27.2] | 48.2 [44.5,52] | 19.2 [16.5,22.3] |
| capable people | 3.8 [2.6,5.4] | 23.4 [20.4,26.7] | 40.7 [37.1,44.5] | 27.6 [24.4,31.1] | 4.5 [3.1,6.4] |
| able to do things | 7.0 [5.4,9.1] | 27.8 [24.6,31.2] | 37 [33.4,40.7] | 24.8 [21.6,28.3] | 3.4 [2.2,5.1] |
|  |  |  |  |  |  |
| ADHD |  |  |  |  |  |
| snap out of it | 37.7 [34.2,41.4] | 39.6 [36.0,43.3] | 16.9 [14.2,20.0] | 5.1 [3.7,7.2] | 0.6 [0.3,1.5] |
| personal weakness | 36.7 [33.3,40.4] | 42.8 [39.2,46.5] | 13.8 [11.3,16.7] | 6.0 [4.4,8.2] | 0.6 [0.3,1.5] |
| not real medical illness | 32.9 [29.5,36.5] | 43.6 [40.0,47.4] | 18.7 [15.9,21.8] | 4.2 [3.0,5.9] | 0.6 [0.2,1.4] |
| dangerous | 32.8 [29.4,36.4] | 40.4 [36.8,44.0] | 22.7 [19.7,26.0] | 4.0 [2.6,6.2] | 0.1 [0.0,0.3] |
| best to avoid | 52.2 [48.5,56.0] | 36.7 [33.2,40.4] | 8.3 [6.4,10.6] | 1.6 [0.8,3.0] | 1.2 [0.5,2.7] |
| unpredictable | 7.7 [5.8,10.2] | 22.5 [19.6,25.7] | 40.6 [37.0,44.3] | 27.2 [24.0,30.6] | 1.9 [1.1,3.4] |
| would not tell | 13 [10.6,15.9] | 39.1 [35.5,42.8] | 30.4 [27.1,34.0] | 14.8 [12.4,17.6] | 2.6 [1.6,4.1] |
| would not employ | 15.8 [13.0,18.9] | 30.5 [27.2,33.9] | 34.1 [30.6,37.7] | 16.6 [14.1,19.5] | 3.0 [2.0,4.7] |
| would not vote for | 16.8 [14.0,20.1] | 24.7 [21.7,28.0] | 27.5 [24.3,30.9] | 23.6 [20.6,26.8] | 7.4 [5.6,9.7] |
| force into treatment | 31.2 [27.8,34.8] | 39.4 [35.9,43.1] | 22.3 [19.2,25.7] | 6.3 [4.6,8.5] | 0.8 [0.4,1.9] |
| persons of worth | 1.1 [0.5,2.3] | 3.1 [2.1,4.5] | 20.5 [17.6,23.8] | 44.4 [40.7,48.1] | 30.9 [27.6,34.5] |
| capable people | 1.6 [0.8,2.9] | 8.0 [6.2,10.3] | 28 [24.8,31.4] | 47.9 [44.2,51.6] | 14.5 [12.0,17.5] |
| able to do things | 1.4 [0.7,2.7] | 17.8 [15.1,20.7] | 26.8 [23.6,30.2] | 41.9 [38.3,45.6] | 12.1 [9.8,14.9] |

Table S2. Social Distance Scale item responses per vignette

|  | Definitely willing  % [95% CI] | Probably willing  % [95% CI] | Probably unwilling  % [95% CI] | Definitely unwilling  % [95% CI] |
| --- | --- | --- | --- | --- |
| Depression |  |  |  |  |
| Move next door | 37.8 [34.3,41.4] | 52.9 [49.2,56.6] | 7.7 [6.0,9.9] | 1.6 [0.8,3.2] |
| Spend evening socialising | 32.6 [29.3,36.1] | 56.8 [53.1,60.4] | 9.4 [7.4,12.0] | 1.2 [0.6,2.4] |
| Make friends with | 32.5 [29.1,36.1] | 57.8 [54.1,61.5] | 9.3 [7.3,11.7] | 0.4 [0.1,1.3] |
| Work closely on a job | 25.2 [22.2,28.6] | 54.3 [50.6,58.0] | 18.2 [15.5,21.3] | 2.2 [1.4,3.7] |
| Marry into family | 19.7 [16.9,22.8] | 50.5 [46.8,54.2] | 24.6 [21.5,27.9] | 5.3 [3.7,7.4] |
| Early schizophrenia |  |  |  |  |
| Move next door | 17.4 [14.7,20.4] | 54.6 [50.7,58.4] | 24.7 [21.4,28.4] | 3.3 [2.2,4.9] |
| Spend evening socialising | 20.5 [17.6,23.7] | 56.4 [52.6,60.2] | 20.4 [17.4,23.9] | 2.7 [1.7,4.2] |
| Make friends with | 17.2 [14.4,20.3] | 56.0 [52.1,59.8] | 24.1 [20.8,27.7] | 2.8 [1.7,4.5] |
| Work closely on a job | 12.8 [10.6,15.5] | 54.0 [50.1,57.8] | 30.1 [26.6,33.8] | 3.1 [2.0,4.8] |
| Marry into family | 7.9 [6.1,10.1] | 40.7 [37.0,44.4] | 44.3 [40.4,48.2] | 7.2 [5.5,9.4] |
| Bipolar disorder |  |  |  |  |
| Move next door | 25.2 [22.2,28.6] | 56.8 [53.1,60.5] | 15.4 [12.9,18.3] | 2.5 [1.6,3.9] |
| Spend evening socialising | 29.0 [25.7,32.5] | 56.0 [52.2,59.6] | 13.1 [10.9,15.6] | 2.0 [1.2,3.5] |
| Make friends with | 24.8 [21.6,28.2] | 59.5 [55.8,63.1] | 14.2 [11.9,16.8] | 1.6 [0.8,3.0] |
| Work closely on a job | 19.0 [16.2,22.1] | 58.0 [54.3,61.6] | 19.2 [16.5,22.3] | 3.8 [2.6,5.5] |
| Marry into family | 16.6 [14.0,19.7] | 45.9 [42.2,49.6] | 30.8 [27.5,34.4] | 6.7 [5.0,8.8] |
| Borderline |  |  |  |  |
| Move next door | 14.6 [12.2,17.4] | 52.4 [48.7,56.1] | 28 [24.8,31.5] | 4.9 [3.5,6.9] |
| Spend evening socialising | 15.1 [12.7,17.8] | 57.2 [53.5,60.8] | 24.5 [21.4,27.8] | 3.3 [2.1,5.1] |
| Make friends with | 12.4 [10.2,15.1] | 55.9 [52.2,59.5] | 27.9 [24.7,31.3] | 3.8 [2.5,5.5] |
| Work closely on a job | 10.6 [8.5,13.1] | 48.9 [45.2,52.7] | 34.9 [31.5,38.6] | 5.5 [4.0,7.6] |
| Marry into family | 7.7 [5.9,9.9] | 33.9 [30.5,37.6] | 43.7 [40.1,47.5] | 14.7 [12.2,17.5] |
| Long-term/untreated schizophrenia |  |  |  |  |
| Move next door | 11.5 [9.2,14.2] | 47.9 [44.1,51.6] | 32.6 [29.1,36.2] | 8.1 [6.2,10.5] |
| Spend evening socialising | 11.8 [9.6,14.6] | 49.3 [45.5,53.0] | 31.0 [27.6,34.5] | 7.9 [6.1,10.3] |
| Make friends with | 9.5 [7.6,11.9] | 48.2 [44.4,51.9] | 36.4 [32.9,40.1] | 5.9 [4.3,8.0] |
| Work closely on a job | 8.7 [6.7,11.1] | 45.5 [41.8,49.2] | 37.7 [34.1,41.4] | 8.1 [6.3,10.5] |
| Marry into family | 6.1 [4.5,8.2] | 29.6 [26.3,33.2] | 45.5 [41.8,49.3] | 18.8 [16.0,22.1] |
| ADHD |  |  |  |  |
| Move next door | 37.8 [34.3,41.5] | 50.0 [46.2,53.7] | 11.3 [9.1,13.9] | 0.9 [0.5,1.8] |
| Spend evening socialising | 34.8 [31.3,38.5] | 53.0 [49.2,56.7] | 10.9 [8.8,13.4] | 1.3 [0.7,2.3] |
| Make friends with | 32.5 [29.0,36.1] | 55.2 [51.4,58.9] | 11.2 [9,13.9] | 1.1 [0.6,2.3] |
| Work closely on a job | 23.3 [20.1,26.7] | 48.1 [44.4,51.9] | 24.6 [21.5,27.9] | 4.0 [2.7,5.8] |
| Marry into family | 26.8 [23.5,30.4] | 43.5 [39.8,47.3] | 25.3 [22.2,28.7] | 4.4 [3.1,6.1] |

We conducted an exploratory factor analysis on the polychoric correlation matrix of the 13 personal stigma items, with principal axis factoring. Visual inspection of the scree plot (Figure S1) and a parallel analysis (Figure S2) indicated a two-factor structure (factor 1 eigenvalue = 4.44, factor 2 eigenvalue = 1.34).

Factor loadings for the two-factor structure are shown in Table S3. Item 7 did not load on either factor and item 11 loaded substantively on both factors. These items were removed to ensure an interpretable factor structure. Based on the content of items in the factors, they were named dangerous/unpredictable/incapable and ‘weak not sick’.

Table S3. Exploratory factor analysis of the personal stigma items (N=5971)^a^

|  |  | Factor 1 (Dangerous/ unpredictable/ incapable) | Factor 2 (Weak not sick) | Communality |
| --- | --- | --- | --- | --- |
| 1 | People with a problem like Sam's could snap out of it if they wanted | -0.05 | **0.81** | 0.63 |
| 2 | A problem like Sam's is a sign of personal weakness. | 0.17 | **0.72** | 0.65 |
| 3 | Sam's problem is not a real medical illness. | -0.14 | **0.80** | 0.56 |
| 4 | People with a problem like Sam's are dangerous. | **0.63** | 0.11 | 0.46 |
| 5 | It is best to avoid people with a problem like Sam's so that you don't develop this problem. | 0.23 | **0.62** | 0.56 |
| 6 | People with a problem like Sam's are unpredictable. | **0.66** | -0.10 | 0.39 |
| 7 | If I had a problem like Sam's I would not tell anyone. | 0.11 | 0.18 | 0.06 |
| 8 | I would not employ someone if I knew they had a problem like Sam's. | **0.73** | 0.07 | 0.58 |
| 9 | I would not vote for a politician if I knew they had suffered a problem like Sam's. | **0.58** | 0.15 | 0.43 |
| 10 | People with a problem like Sam's should be forced into treatment even if they don't want to. | **0.47** | 0.12 | 0.28 |
| 11 | People with a problem like Sam's are able to do things as well as most other people. | **-0.34** | **-0.41** | 0.40 |
| 12 | I see people with a problem like Sam's as capable people | **-0.68** | 0.01 | 0.45 |
| 13 | I feel people with a problem like Sam's are persons of worth, at least on an equal basis with others. | **-0.62** | 0.17 | 0.32 |
|  |  |  |  |  |
|  | Variance | 3.76 | 3.36 |  |
|  | Proportion of common variance | 0.68 | 0.61 |  |

1. Principal factor extraction with oblique (direct oblimin) rotation. Factor loadings above .3 are in bold. The correlation between factors was .43.

Figure S1. Scree plot of eigenvalues from the factor analysis, used to guide factor retention


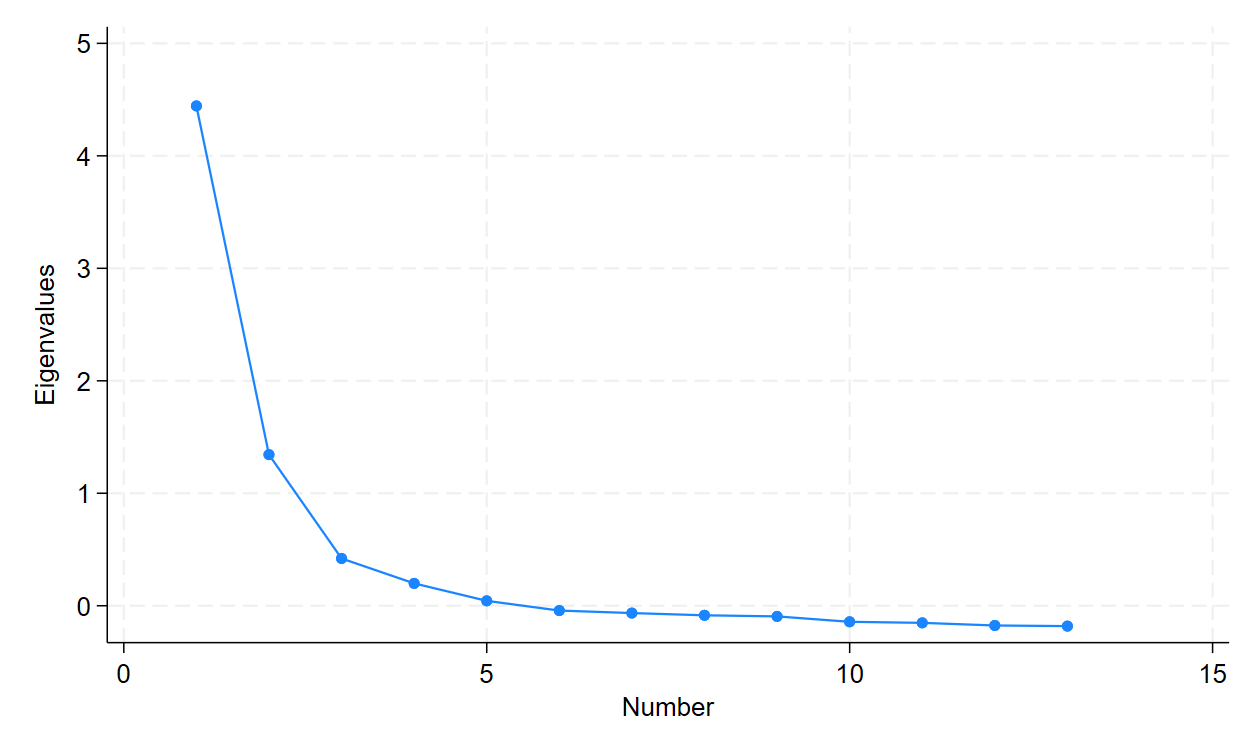


Figure S2. Parallel analysis scree plot. Observed eigenvalues (red) exceeded random eigenvalues (blue) for the first two factors, suggesting a two-factor solution.


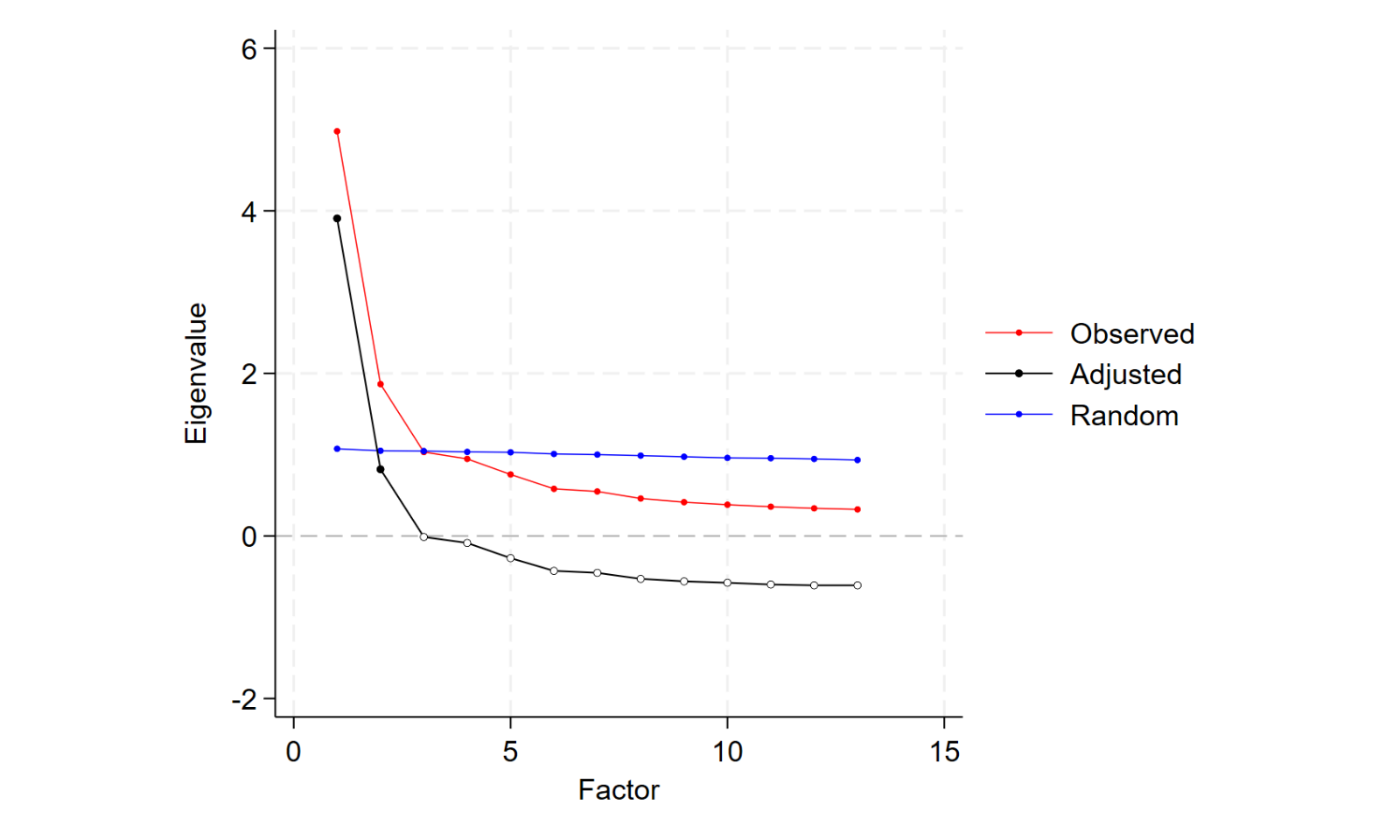


**Checklist for Reporting Of Survey Studies (CROSS)**

| **Section/topic** | **Item** | **Item description** | **Reported on page #** |
| --- | --- | --- | --- |
| **Title and abstract** | | |  |
| Title and abstract | 1a | State the word “survey” along with a commonly used term in title or abstract to introduce the study’s design. | 1 |
|  | 1b | Provide an informative summary in the abstract, covering background, objectives, methods, findings/results, interpretation/discussion, and conclusions. | 2-3 |
| **Introduction** | | |  |
| Background | 2 | Provide a background about the rationale of study, what has been previously done, and why this survey is needed. | 4-5 |
| Purpose/aim | 3 | Identify specific purposes, aims, goals, or objectives of the study. | 5 |
| **Methods** | | |  |
| Study design | 4 | Specify the study design in the methods section with a commonly used term (e.g., cross-sectional or longitudinal). | 5 |
|  | 5a | Describe the questionnaire (e.g., number of sections, number of questions, number and names of instruments used). | 6-7 |
| Data collection methods | 5b | Describe all questionnaire instruments that were used in the survey to measure particular concepts. Report target population, reported validity and reliability information, scoring/classification procedure, and reference links (if any). | 6-7, supplementary material |
|  | 5c | Provide information on pretesting of the questionnaire, if performed (in the article or in an online supplement). Report the method of pretesting, number of times questionnaire was pre-tested, number and demographics of participants used for pretesting, and the level of similarity of demographics between pre-testing participants and sample population. | N/A |
|  | 5d | Questionnaire if possible, should be fully provided (in the article, or as appendices or as an online supplement). | 6-7 |
| Sample characteristics | 6a | Describe the study population (i.e., background, locations, eligibility criteria for participant inclusion in survey, exclusion criteria). | 5-6 |
|  | 6b | Describe the sampling techniques used (e.g., single stage or multistage sampling, simple random sampling, stratified sampling, cluster sampling, convenience sampling). Specify the locations of sample participants whenever clustered sampling was applied. | 6 |
|  | 6c | Provide information on sample size, along with details of sample size calculation. | 6 |
|  | 6d | Describe how representative the sample is of the study population (or target population if possible), particularly for population-based surveys. | 7-8 |
| Survey  administration | 7a | Provide information on modes of questionnaire administration, including the type and number of contacts, the location where the survey was conducted (e.g., outpatient room or by use of online tools, such as SurveyMonkey). | 6 |
|  | 7b | Provide information of survey’s time frame, such as periods of recruitment, exposure, and follow-up days. | 6 |
|  | 7c | Provide information on the entry process:  –>For non-web-based surveys, provide approaches to minimize human error in data entry.  –>For web-based surveys, provide approaches to prevent “multiple participation” of participants. | 6 |
| Study preparation | 8 | Describe any preparation process before conducting the survey (e.g., interviewers’ training process, advertising the survey). | N/A |
| Ethical considerations | 9a | Provide information on ethical approval for the survey if obtained, including informed consent, institutional review board [IRB] approval, Helsinki declaration, and good clinical practice [GCP] declaration (as appropriate). | 8 |
|  | 9b | Provide information about survey anonymity and confidentiality and describe what mechanisms were used to protect unauthorized access. | 6 |
| Statistical  analysis | 10a | Describe statistical methods and analytical approach. Report the statistical software that was used for data analysis. | 7-8 |
|  | 10b | Report any modification of variables used in the analysis, along with reference (if available). | N/A |
|  | 10c | Report details about how missing data was handled. Include rate of missing items, missing data mechanism (i.e., missing completely at random [MCAR], missing at random [MAR] or missing not at random [MNAR]) and methods used to deal with missing data (e.g., multiple imputation). | 8 |
|  | 10d | State how non-response error was addressed. | 7-8 |
|  | 10e | For longitudinal surveys, state how loss to follow-up was addressed. | N/A |
|  | 10f | Indicate whether any methods such as weighting of items or propensity scores have been used to adjust for non-representativeness of the sample. | 7-8 |
|  | 10g | Describe any sensitivity analysis conducted. | N/A |
| **Results** | | |  |
| Respondent characteristics | 11a | Report numbers of individuals at each stage of the study. Consider using a flow diagram, if possible. | 6 |
|  | 11b | Provide reasons for non-participation at each stage, if possible. | N/A |
|  | 11c | Report response rate, present the definition of response rate or the formula used to calculate response rate. | 6 |
|  | 11d | Provide information to define how unique visitors are determined. Report number of unique visitors along with relevant proportions (e.g., view proportion, participation proportion, completion proportion). | N/A |
| Descriptive  results | 12 | Provide characteristics of study participants, as well as information on potential confounders and assessed outcomes. | Table 2 |
| Main findings | 13a | Give unadjusted estimates and, if applicable, confounder-adjusted estimates along with 95% confidence intervals and p-values. | Table S1, S2 |
|  | 13b | For multivariable analysis, provide information on the model building process, model fit statistics, and model assumptions (as appropriate). | 8 |
|  | 13c | Provide details about any sensitivity analysis performed. If there are considerable amount of missing data, report sensitivity analyses comparing the results of complete cases with that of the imputed dataset (if possible). | N/A |
| **Discussion** | | |  |
| Limitations | 14 | Discuss the limitations of the study, considering sources of potential biases and imprecisions, such as non-representativeness of sample, study design, important uncontrolled confounders. | 10-11 |
| Interpretations | 15 | Give a cautious overall interpretation of results, based on potential biases and imprecisions and suggest areas for future research. | 11 |
| Generalizability | 16 | Discuss the external validity of the results. | 10 |
| **Other sections** | | |  |
| Role of funding source | 17 | State whether any funding organization has had any roles in the survey’s design, implementation, and analysis. | Online submission |
| Conflict of interest | 18 | Declare any potential conflict of interest. | Online submission |
| Acknowledgements | 19 | Provide names of organizations/persons that are acknowledged along with their contribution to the research. | Online submission |
